# How glycobiology can help us treat and beat the COVID-19 pandemic

**DOI:** 10.1016/j.jbc.2021.100375

**Published:** 2021-02-04

**Authors:** Ricardo D. Lardone, Yohana C. Garay, Pedro Parodi, Sofia de la Fuente, Genaro Angeloni, Eduardo O. Bravo, Anneke K. Schmider, Fernando J. Irazoqui

**Affiliations:** 1 Centro de Investigaciones en Química Biológica de Córdoba, CIQUIBIC, CONICET and Departamento de Química Biológica Ranwel Caputto, Facultad de Ciencias Químicas, Universidad Nacional de Córdoba, Ciudad Universitaria, Córdoba, Argentina; 2 Medicina Interna, Nuevo Hospital San Roque, Ministerio de Salud de la Provincia de Córdoba, Córdoba, Argentina; 3 Klinik für Kinder- und Jugendpsychiatrie und Psychotherapie, Psychiatrische Klinik Lüneburg, Lüneburg, Germany

**Keywords:** COVID-19, glycobiology, SARS-CoV-2, potential therapies, FDA-approved drugs, ACE2, angiotensin-converting enzyme 2, DC-SIGN, dendritic cell-specific ICAM-3-grabbing non-integrin, Gal-3, galectin 3, HS, heparan sulfate, IMP, importin, MBL, mannose-binding lectin, MGL, macrophage galactose lectin, RBD, receptor-binding domain, SARS-CoV-2, severe acute respiratory syndrome coronavirus 2

## Abstract

Severe acute respiratory syndrome coronavirus 2 (SARS-CoV-2) emerged during the last months of 2019, spreading throughout the world as a highly transmissible infectious illness designated as COVID-19. Vaccines have now appeared, but the challenges in producing sufficient material and distributing them around the world means that effective treatments to limit infection and improve recovery are still urgently needed. This review focuses on the relevance of different glycobiological molecules that could potentially serve as or inspire therapeutic tools during SARS-CoV-2 infection. As such, we highlight the glycobiology of the SARS-CoV-2 infection process, where glycans on viral proteins and on host glycosaminoglycans have critical roles in efficient infection. We also take notice of the glycan-binding proteins involved in the infective capacity of virus and in human defense. In addition, we critically evaluate the glycobiological contribution of candidate drugs for COVID-19 therapy such as glycans for vaccines, anti-glycan antibodies, recombinant lectins, lectin inhibitors, glycosidase inhibitors, polysaccharides, and numerous glycosides, emphasizing some opportunities to repurpose FDA-approved drugs. For the next-generation drugs suggested here, biotechnological engineering of new probes to block the SARS-CoV-2 infection might be based on the essential glycobiological insight on glycosyltransferases, glycans, glycan-binding proteins, and glycosidases related to this pathology.

During the last months of 2019, a severe acute respiratory syndrome coronavirus 2 (SARS-CoV-2) emerged in Wuhan (province of Hubei, China) causing a highly transmissible infectious illness that the World Health Organization officially named as “coronavirus disease 2019,” or COVID-19 (1). The rapid dissemination of this virus critically affected health, social behaviors, and economy of all countries around the world. At present, the American continent is the world’s region with the highest number of infected and deceased population. The viral dissemination occurs through infected persons who transport and spread infective virus particles to the environment. SARS-CoV-2 is critically dependent on host cells for RNA replication, protein translation, and progeny virus particle assembly for next dissemination (Fig. 1). Thus, the released virus progeny can infect neighboring cells in the same patient or can be transferred to other persons through saliva microdroplets (3). SARS-CoV-2 infectivity declines approximately 10 days after onset of symptoms (Fig. 2), but the virus can still be detected up to 37 days later in some patients (5). COVID-19 evolution depends on the cumulative exposure dose to the virus, the efficacy of host innate immunity, and the achievement of protective adaptive immune response. Most frequent complications were acute respiratory distress syndrome, acute cardiac and kidney injury, sepsis, and secondary infection. Death occurred 18.5 (15.0–22.0) days after disease onset in the case of patients dying from COVID-19 (6). Older age, comorbidities (including hypertension, diabetes, cardiovascular disease, chronic lung disease, and cancer), and secondary infections are associated with higher mortality. COVID-19 has been proposed as a viral multisystem disease, with dominant vascular pathology (7). In this complex scenery, we need to understand the biology of the virus and think about ways to quickly treat it. Glycobiology is the study of biosynthesis, structure, function, and evolution of glycans distributed in nature and the proteins that recognize them. Glycans have critical roles in the pathobiology of viral infections. Glycoconjugates are involved in the folding, stability, and protection of viral proteins, as well as in the specific cellular tropism of virus (8). The surface of the viral cover has specific proteins involved in the process of binding to host cells for viral entry (9). Glycans also work as shielding of specific peptide epitopes, protecting them from antibody neutralization. This immunogenic camouflage has been also described for other coronaviruses (10, 11, 12). Viral glycoproteins of HIV-1 Env (13), influenza hemagglutinin (14, 15), and LASV GPC (16) also shield the receptor-binding site with glycans. Thus, glycosylation can facilitate the virus evasion from the innate and adaptive immune responses (17). In this review, we explore the glycobiology related to SARS-CoV-2, from virus to host to new ideas for treatments.

**Figure 1:**
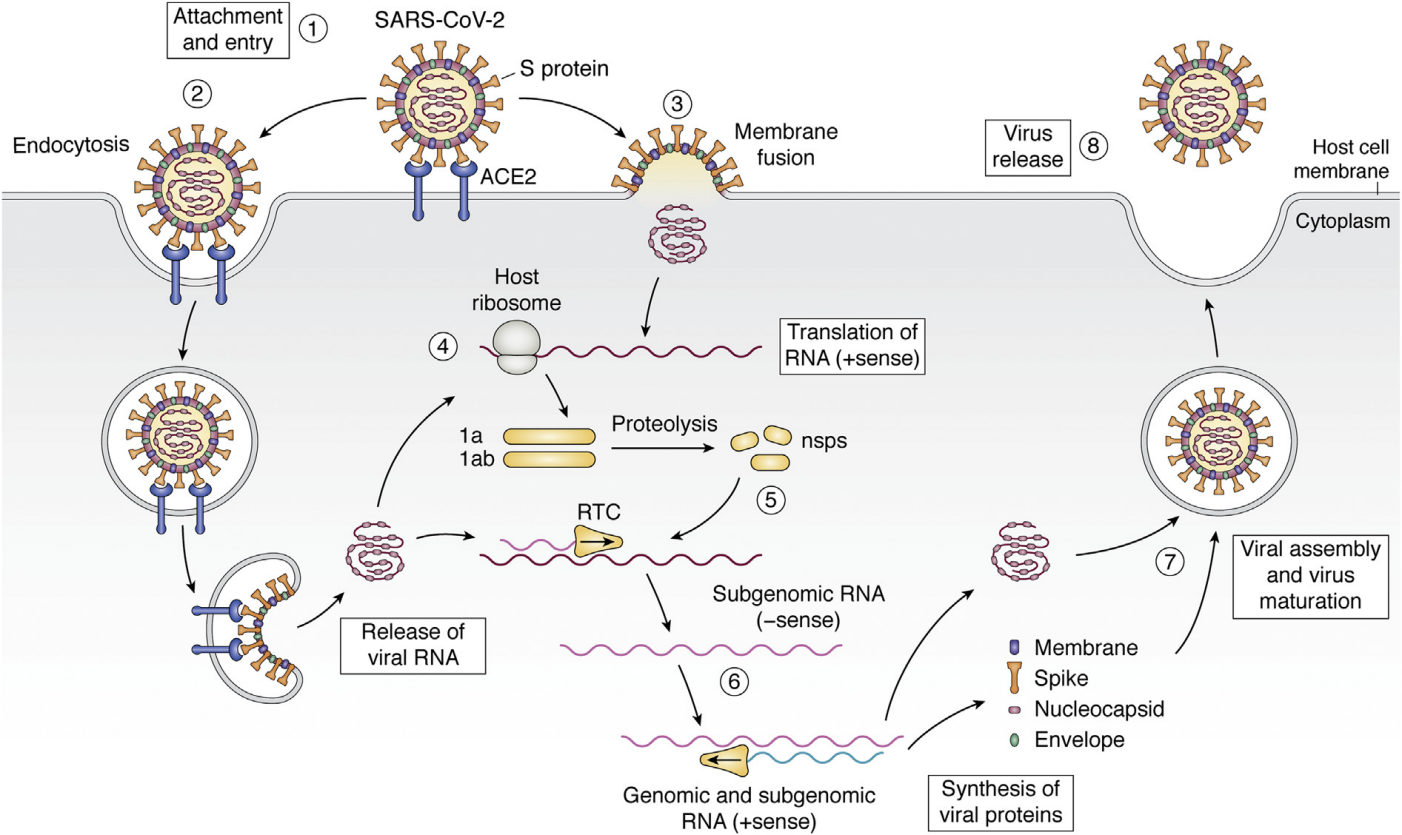
**Infective life cycle of SARS-CoV-2**. The interaction between the S protein and ACE2 receptor allows the attachment of the virus to the host cell (1). After that, the entry can be achieved by endocytosis (2) or by fusion of the viral and cellular membranes (3). Once inside, the viral RNA begins to be translated for producing the 1a and 1ab proteins (4). These proteins undergo subsequent proteolysis to produce nonstructural proteins (nsps), which complex thereafter to form the replicase-transcriptase complex (RTC) (5). The RTC is in charge of synthesizing the new viral RNA (- sense) and the structural viral proteins; both genomic and subgenomic RNAs are produced through negative-strand intermediates (6). At the end of the process, the assembly of the viral particle occurs (7) and the virus is released by exocytosis (8) (2).

**Figure 2:**
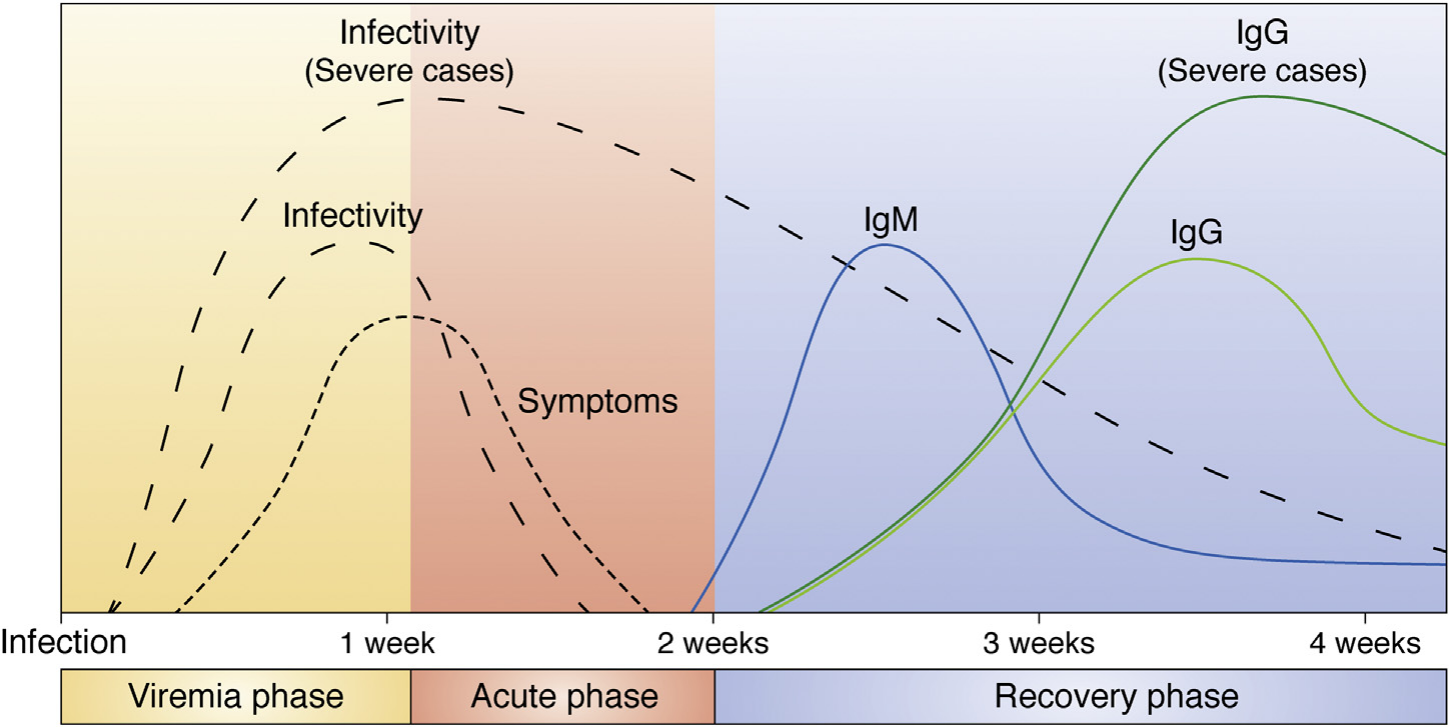
**Representative scheme of COVID-19 pathogenesis**. Trajectory of SARS-CoV-2 shedding; symptoms and adaptive immune response of patients without or with minor airways difficulties and with severe disease (4).

## Infection of SARS-CoV-2

**Figure 3:**
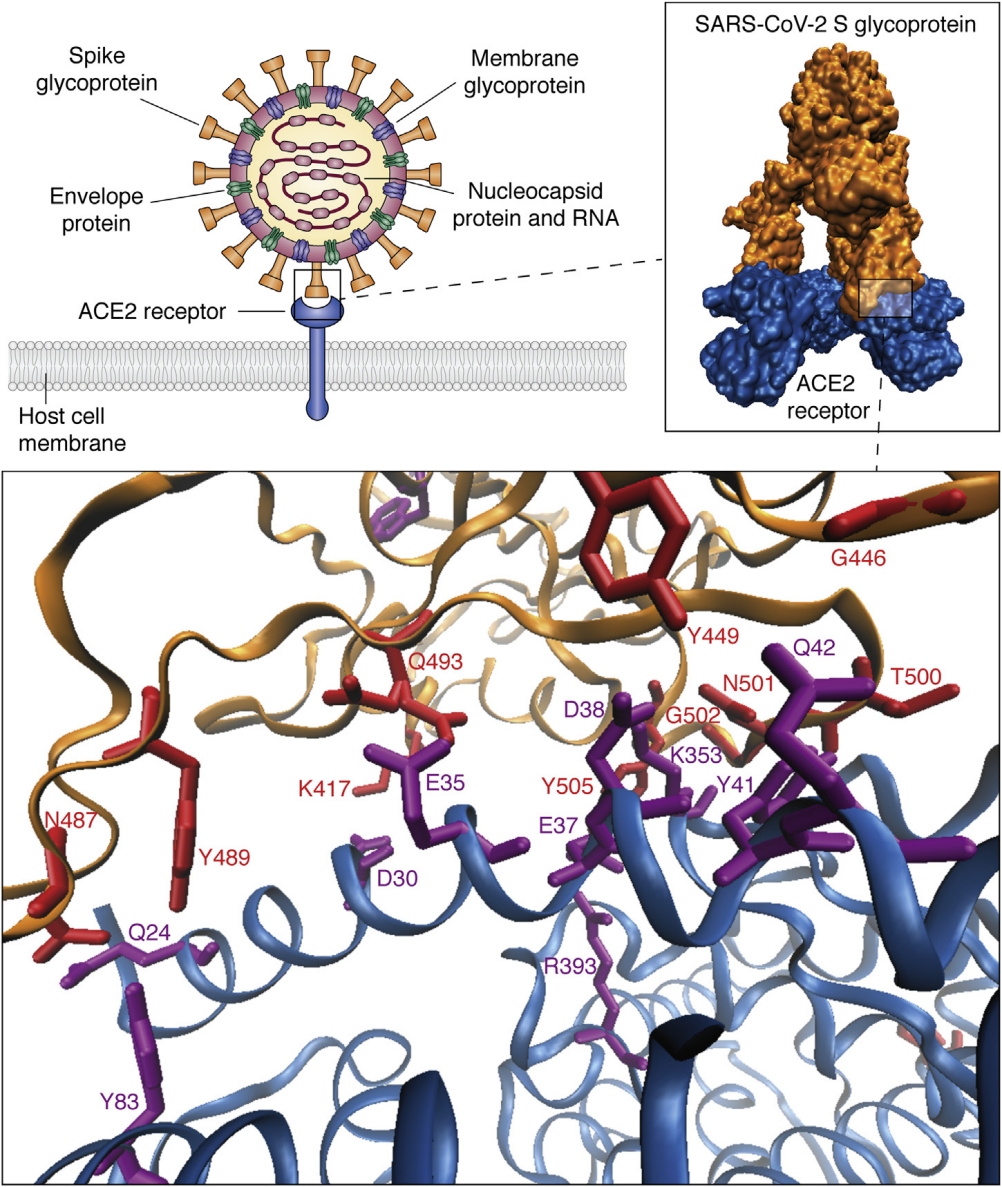
**SARS-CoV-2 S glycoprotein–ACE2 interaction.** Viral particles attach to the cell membrane through a very specific interaction between the viral spike glycoprotein and ACE2 on host cell surface. PDB models (6M0J and 7A98) were retrieved from (21).

**Figure 4:**
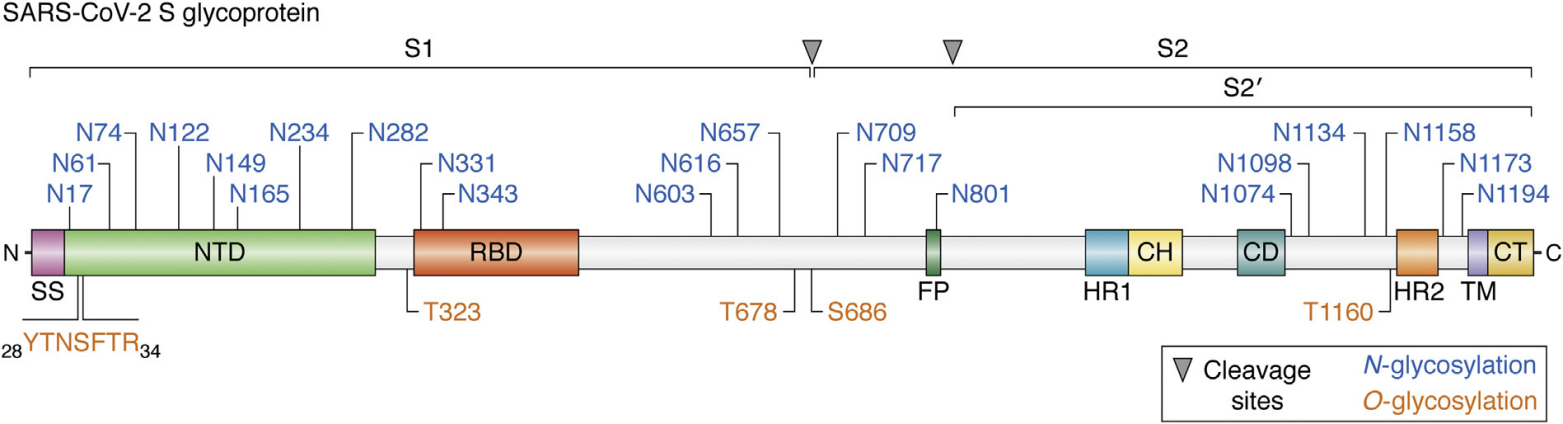
**Schematic of SARS-CoV-2 S glycoprotein domains, subunits, and N-/O-glycan sites.** The different regions and components are represented along the protein backbone (*light blue*). Protein domains (shown in *different colors*) are as follows: SS, signal sequence; NTD, N-terminal domain; RBD, receptor-binding domain; FP, fusion peptide; HR1, heptad repeat 1; CH, central helix; CD, connector domain; HR2, heptad repeat 2; TM, transmembrane domain; CT, cytoplasmic tail. Sites for N-glycosylation (*blue*) and O-glycosylation (*orange*) are indicated with their specific amino acid position. The cleavage sites for S1/S2 and S2’ proteases are shown on top (10, 23, 24).

Viral SARS-CoV-2 particles (60–140 nm) contain single positive-stranded ribonucleic acid (RNA) from 26 to 32 kb in length. Viral sequencing demonstrated six major open-reading frames and a number of other accessory genes encoding spike protein, 3-chymotrypsin-like cysteine protease (also named main protease), papain-like protease, and RNA-dependent RNA polymerase (18, 19). The SARS-CoV-2 outer membrane spike (S) glycoprotein is a homotrimer of the viral surface, giving the virus a halo- or crown-like appearance, common to members of the coronavirus family. S glycoprotein, highlighted in this review by its critical role for cell adhesion and virulence, is the prime interacting protein with host cell target receptor angiotensin-converting enzyme 2 (ACE2) (20) (Fig. 3). ACE2 is a type 1 transmembrane protein expressed by host epithelial cells. ACE2 shows medium expression levels in normal human lung, colon, liver, bladder, and adrenal gland tissues and high expression levels in small intestine, testis, kidney, heart, thyroid, and adipose tissue (22). The recognition of ACE2 occurs through the receptor-binding domain (RBD) of SARS-CoV-2 S glycoprotein (Figs. 3 and 4). RBD (aa329–521) is located in the S1 domain of SARS-CoV-2 S glycoprotein, which shows ∼55% identity between SARS-CoV-2 and SARS-CoV. RBD has a hinge-like dynamic movement to enhance the binding of RBD with ACE2 (25). Mutations in the SARS-CoV RBD led to acquisition of a furin cleavage site that has given SARS-CoV-2 virus high infectivity, high pathogenicity, and an increased ability for cross-species and human-to-human transmission (26). After the S glycoprotein RBD–ACE2 interaction, the cleavage of furin site by TGRBSS2 serine protease yields S1 (aa13–685) and S2 (aa686–1273) subunits on S glycoprotein (Fig. 4), enhancing the ability of the virus to internalize into host cells (27). The S1 subunit of S glycoprotein contains the RBD, which allows coronavirus to bind to the peptidase domain of ACE2, whereas the S2 subunit plays a key role in membrane fusion for viral infection.

## Glycobiology of SARS-CoV-2 infection

SARS-CoV-2 S glycoprotein is extensively glycosylated, showing 22 N-glycan residues when the protein was expressed in human HEK-293 cells (10, 23) (Fig. 4). The N165, N234, N331, and N343 glycosylation sites neighboring to RBD shield this critical region for immune response recognition. Beyond shielding, N165 and N234 glycans seem to have an additional role as modulators of the RBD conformational plasticity: their presence stabilizes the RBD “up” conformation (which allows binding to ACE2), whereas deletion of these glycans through N165A and N234A mutations significantly reduced binding to ACE2 as a result of the RBD conformational shift toward the “down” state, not accessible to ACE2 (28).

The content of oligomannose-type glycans in S protein represents the 28% of total molecular weight (lower than for other viral glycoproteins), and the N234 glycosite adjacent to RBD displays Man_5_-GlcNAc_2_ oligomannose-type glycans (10). N165, N331, and N343 glycosites report complex-type N-glycans, highlighting fucosylated and branched two- and three-antennary N-glycans. The N343 glycosite contains N-glycans with 98% fucosylation (10). In addition, O-glycans observed on viral proteins have been suggested to play key roles in the biological activity of viral proteins (29). The presence of O-GalNAc glycosylation at T323 and the possible glycosylation at S325 were previously determined (23). In addition, O-GalNAc residues in the N-terminal domain peptide _28_YTNSFTR_34_ and in T678, S686, and T1160 residues of SARS-CoV-2 S glycoprotein were described (Fig. 4), along with identification of O-GalNAc glycans such us Tn, core 1, mono- and di-sialyl core 1, and sialylated core 2 structures (24). The O-GalNAc glycans located in the hinge region of RBD (T323 and S325) and those neighboring to furin cleavage site (S686) could play a critical role in viral binding to ACE2 receptors and in the membrane fusion required for viral infection, respectively.

In a similar way, glycosylation of ACE2 receptor also has influence on virus attachment and infectivity. Glycosylation at N90 partly interferes with the RBD-ACE2 interaction, whereas any mutation at N90 or T92 removing the canonical N-glycosylation sequon at N90 renders the unglycosylated variant more prone to interact with RBD (30). Of interest, the glycan at N322 has an opposite behavior: it interacts tightly with RBD, strengthening the ACE2–RBD complex (31). In addition, intermolecular glycan–glycan interactions have been predicted between the glycan at N546 of ACE2 and those at the N74 and N165 residues from S glycoprotein (32). Thus, changes in glycan occupancy or processing at these ACE2 sites could modify the affinity of the SARS-CoV-2–ACE2 interaction, modulating infectivity. Regarding O-glycosylation of ACE2, T730 is the only residue fully glycosylated (33). Located in the juxtamembrane region (just outside the cell membrane and neighboring the cleavage site for the ADAM17-induced ectodomain shedding of ACE2), this bulky and hydrophilic T730 glycosylation could influence the dimerization, presentation, and shedding of ACE2 on the cell surface (33).

Carbohydrate-binding proteins are molecules with the ability to decode glycan information related to several biological functions (34). Multiple viruses are enveloped with carbohydrate-binding proteins involved in relevant functions such as viral infectivity: herpes simplex virus (35), human immunodeficiency virus (36) and human coronavirus NL63 (37) interact with heparan sulfates (HSs). The SARS-CoV-2 S glycoprotein was also reported to have this glycan-binding property. Recently, two different glycan-binding pockets were described in the SARS-CoV-2 S glycoprotein homotrimer. The first glycan-binding pocket is a cavity located in the S2 subunit of SARS-CoV-2 S glycoprotein homotrimer that binds chitosan (GlcN-GlcNAc)_n_, a polysaccharide obtained from chitin polymer (38). The structural orientation of these S2 subunit domains would indicate that they form a large cavity using three chains from a homotrimer S glycoprotein to bind chitosan with high affinity (38). The slight movement observed in the homotrimer cavity and the structural orientations suggest that it could work as a “bouncing spring.” When the S glycoprotein interacts with the ACE2 receptor, this bouncing spring movement may be important in the fusion of the virion with the host cell membrane (38). The other glycan-binding pocket, described as a heparin/HS-binding site adjacent to the domain that binds to ACE2, suggests that RBD could simultaneously interact with HS oligosaccharide and ACE2 protein of host cells during the viral infection (39). The interaction of HS oligosaccharide with SARS-CoV-2 RBD amino acids (R346, F347, S349, N354, R355, K444, G447, Y449, Y451, and R466) is mainly conserved with respect to SARV-CoV-1, except for K444T and N354E. These amino acid substitution changes may mediate the enhanced interaction with HS of SARS-CoV-2 compared with SARS-CoV-1 (39). In addition, results with native SARS-CoV-2 demonstrated that cellular HS is required for effective infection and this binding to HS occurs in a cooperative manner with ACE2 protein. Thus, the HS-binding ability of SARS-CoV-2 S glycoprotein demonstrated to be a crucial viral component for the infection. Elucidation of the pathogenic mechanism of SARS-CoV-2 infection is critical to uncover and design therapeutic drugs.

## Glycobiological human defense to SARS-CoV-2 infection

### First line of defense: host glycans

The O-GalNAc glycans of the secreted mucins are essential to hydrate and protect the epithelial cells in the respiratory, gastrointestinal, and genitourinary tracts and those of the eyes. Mucins also trap microorganisms *via* their O-glycans, thus playing an important physiological process in removing microbes and particles trapped in mucus (40). Mucins of saliva form a coat on hard and soft tissues that, by specific binding and aggregation mechanisms, block the adherence of microorganisms to oral surfaces facilitating the clearance of microorganisms (41). Mucins also have a central role in respiratory tract health by providing a physical barrier and cleaning for pathogens, thus influencing the morbidity and mortality of patients with lung diseases (42).

### Second line of defense: soluble glycan-binding proteins

Innate immunity is the second line of defense against the SARS-CoV-2 virus, and this outcome decides the natural history of the disease. Human antiviral innate immunity is partially based on soluble elements such as lectins, the complement system, interferons, cytokines, the coagulation system, and natural antibodies (IgM, IgA, and IgG) (43, 44). If the SARS-CoV-2 can overcome the innate immunity and arrive to lower airways and alveoli during the early phase of infection, the pneumonia complications by viral infection are higher (45). The soluble serum protein mannose-binding lectin (MBL), with a central role in innate immunity (46), recognizes and binds to terminal mannose glycans of microorganisms, enhancing the opsonophagocytosis, activating the complement pathway, and modulating inflammation as a member of the second line of defense (45). Evidence suggests that MBL may protect in the early stages of SARS-CoV-2 infection. MBL could also be involved in the recognition of oligomannose-type glycans exposed in the SARS-CoV-2 S protein (10) representing a susceptibility factor for the acquisition of this viral infection (47). Serum MBL levels are enhanced in children compared with adults (over 20 years) and decline with age (48). Reduced MBL concentrations in elder persons are coincident with higher SARS-CoV-2 susceptibility suggesting a protagonist role in the antiviral defense.

Natural anti-glycan antibodies are relevant soluble components of innate immunity against viral infections, where the IgM isotype has a central role but IgG and IgA are also are relevant (49, 50). Natural anti-glycan IgM antibodies occur in neonates as a response to bacterial colonization, reaching IgM concentration levels similar to those in adults relatively early in life (51). This immune response is addressed to microorganism antigens and autoantigens such as ABO blood groups (A: GalNAcα3(Fucα2)Galβ-; B: Galα3(Fucα2)Galβ- and O: Fucα2Galβ-), stabilizing maximum antibody concentrations from childhood on. Natural IgM antibodies bind, neutralize, and clear certain viruses such as vesicular stomatitis virus, lymphocytic choriomeningitis virus, and influenza virus (52, 53). Similarly, natural IgG antibodies also contribute immunity against some viruses like influenza, vesicular stomatitis virus, and uveitis-related lymphotropic virus type 1 (54). Natural anti-glycan IgG antibodies are maintained with age, whereas anti-glycan IgM antibodies decrease in advanced age (55). In agreement, the B-1 cells responsible for IgM production are reduced with advancing age (56). As natural anti-glycan IgM antibodies decline with age, SARS-CoV-2 increases the infection incidence and mortality. A correlation between blood group ABO and SARS-CoV-2 infectivity was also reported (57). Blood group O individuals were more resistant to SARS-CoV infection than blood group A individuals, and this ability was observed with higher (1:256) anti-blood group A antibody titers. In line with these observations, patients with COVID-19 with blood group A or AB were at an increased risk for requiring mechanical ventilation and continuous renal replacement therapy compared with those with blood group O or B (58)

### Third line of defense: cellular glycan-binding proteins

Another member of innate immunity that plays an essential role in viral defense is dendritic cell-specific ICAM-3-grabbing non-integrin (DC-SIGN) lectin, expressed in immature dendritic cells from dermis, lymph nodes, and tonsils (59). DC-SIGN interacts mainly with nonreducing terminal Man and Fuc, and also with Glc, GlcNAc, and ManNAc, reporting a ligand binding promiscuity (60). The attachment of more nonreducing Man or GlcNAc residues tends to enhance DC-SIGN recognition in many cases, but the observed affinity highly depends on the overall glycan geometry (61). DC-SIGN recognizes the SARS-CoV-2 spike protein in a glycan-dependent manner (24). This binding, characterized by affinity in the picomolar range, is consistent with the presence of terminal oligomannose- and complex-type N-glycans in SARS-CoV-2 spike protein.

The human macrophage galactose lectin (MGL) is a calcium-dependent transmembrane member of group II of C-type lectins (62). MGL is mainly expressed in tolerogenic macrophages and dendritic cells undertaking subsequent T cell downregulation inducing apoptosis of effector T cells (63). The role of MGL is associated with protection in persistent inflammation and autoimmune diseases, preventing excessive tissue damage, and allowing tissue remodeling. The MGL structure displays the characteristic QPD motif at the long loop region, typically associated to galactose (Gal/GalNAc) specificity (61). MGL is mainly α-GalNAc specific: interaction with O-GalNAc glycan (GalNAcαO-Ser/Thr) was confirmed, whereas nonreducing terminal α/β-Gal residues are rarely recognized (64). MGL also reports a fine specificity for GalNAc-containing structures conditioned by the neighboring carbohydrate. Gao *et al*. (2020) described the SARS-CoV-2 S protein recognition by MGL, demonstrating that N-glycans and O-GalNAc glycans are involved in the interaction (24).

## Glycobiological contribution on potential candidate molecules for COVID-19 therapy

Emergency caused by SARS-CoV-2 requires urgent and creative therapeutic strategies to prevent and treat this infectious disease that has been producing high number of deaths across the globe. The relevant roles of glycocalyx (glycan pericellular matrix environing the plasma membrane) as mediator in cellular protection and communication with its surroundings expose attractive therapeutic opportunities. Glycobiological strategies may contribute to find an effective therapeutic way for this virulent disease. Here we describe different approaches involving several molecules related to the field of glycobiology that may be considered for SARS-CoV-2 treatment. All suggested ways to COVID-19 therapy must be validated with clinical trials previous to their pharmacotherapy application.

The ideal solution for infectious disorders is the development of vaccines. This approach is currently applied for prevention of viral diseases such as flu (65), hepatitis (66), polio, measles, mumps, varicella, and rubella (67). Antibodies specifically addressed to critical viral proteins that bind host cells prevent the primary virus–cell interaction, thus impeding the viral entry for replication. Immunogenic viral proteins or peptide domains with critical role in the virus–host cell interaction are used for vaccines. In SARS-CoV-2 vaccine it should be logical to consider the RBD of S glycoprotein as a primary target. Several advanced or approved vaccines purpose RBD as immunogen (Anhui Zhifei Longcom Biopharmaceutical) or a target containing RBD such as full-length S glycoprotein (AstraZeneca/Oxford, CanSino, Gamaleya, Janssen, Novavax, Moderna, and Pfizer/BioNTech) (68). This viral RBD has the peculiarity to be shielded by glycans (10), thus reducing the feasibility of proper immunogenicity for the vaccine purpose. Another interesting option is to consider these viral glycans as a target immunogen where the vaccine should be addressed to. In this case it is critical to define a glycan structure (to be used as immunogen) that must be present in the virus and absent in human host (69). Potential glycan structures could be molecules related to the biantennary complex N-glycan with multiple GlcNAc terminals such as GlcNAc_2-4_-Man_3_-GlcNAc_2_-Asn (Table 1). These terminal structures were described as present in N74, N149, N282 of the S1 subunit and N801, N1074, and N1098 of the S2 subunit of SARS-CoV-2 S glycoprotein (23). Also, N234 glycosite adjacent to RBD exposes Man_5_-GlcNAc_2_ oligomannose-type glycans that may be an attractive target for vaccine. In humans, these terminal structures are cryptic N-glycan residues hidden by additional sugar elongation. Although active immunization using different molecules formulated as vaccines is the recommended option for prevention of viral disease, this strategy is not suitable for treatment of patients already infected with SARS-CoV-2. The active immunization approach with vaccines requires several days to develop a proper adaptive immunity to protect the patient from the virus. Passive immunization is the therapeutic strategy using human plasma of people after they overcame the COVID-19 disease: this treatment supplies the patients with already made immune molecules for a fast immune response addressed to the viral particles. Also, in the passive immunization strategy specific human antibodies addressed to O-GalNAc glycans such as GalNAcαO-Ser/Thr (Tn antigen), Galβ3GalNAcαO-Ser/Thr (T antigen), and Neu5Acα3Galβ3GalNAcαO-Ser/Thr (sialyl T antigen) could be used. These O-GalNAc residues were described in the N-terminal domain peptide _28_YTNSFTR_34_, T323 (RBD hinge) and T678 in the S1 subunit, and S686 in the S2 subunit of SARS-CoV-2 spike glycoproteins (24). Tn, T, and sialyl T antigens are cryptic human residues in healthy people, and these anti-O-GalNAc glycan antibodies are commonly present in normal human sera where they can be purified from (70). The use of human anti-Tn, -T and -sialyl T antibodies is an original approach that should also be considered as passive immunotherapy for COVID-19 disease (Table 1). These natural anti-glycan antibodies are relevant components of innate immunity against cancer cells, where the IgM isotype has predominant participation (70). Comorbidity of cancer disease and SARS-CoV-2 infection is often a cause of death (7).

**Table 1:** Potential candidate drugs to COVID-19 prevention and/or treatment^a^

Mechanism of action	Molecular target	Drug	Chemical structure (nature of molecule)	Reference
Active immunization to induce antibodies able to block S glycoprotein	GlcNAc_2_-Man_3_-GlcNAc_2_-Asn on S glycoprotein	GlcNAc_2_-Man_3_-GlcNAc_2_-carrier	https://pubchem.ncbi.nlm.nih.gov/compound/10148744section=2D-Structure (Glycan immunogen)^b^	(23, 24, 69)
	Man_5_-GlcNAc_2_-Asn on S glycoprotein	Man_5_-GlcNAc_2_-carrier	https://pubchem.ncbi.nlm.nih.gov/compound/25229604section=Structures (Glycan immunogen)^b^	(23, 24, 69)
Passive immunization with antibodies able to block S glycoprotein	GalNAc-Ser/Thr (Tn antigen) on S glycoprotein	Human polyclonal anti-Tn antibody	https://pubchem.ncbi.nlm.nih.gov/compound/447272section=2D-Structure (Glycan target)^b^	(23, 24, 70)
	Gal-GalNAc-Ser/Thr (T antigen) on S glycoprotein	Human polyclonal anti-T antibody	https://pubchem.ncbi.nlm.nih.gov/compound/441248section=2D-Structure (Glycan target)^b^	(23, 24, 70)
Protein replacement with recombinant protein able to block S glycoprotein	Man_5_-GlcNAc_2_-Asn on S glycoprotein	Recombinant human mannose-binding lectin	https://pubchem.ncbi.nlm.nih.gov/compound/25229604section=Structures (Glycan target)^b^	(71, 72)
Galectin-3 inhibition	Carbohydrate-recognition domain of galectin-3	GB1107	https://pubchem.ncbi.nlm.nih.gov/compound/122443390section=2D-Structure (Drug)^b^	(73, 74, 75, 76)
		TD139^c^	https://pubchem.ncbi.nlm.nih.gov/compound/73774610section=2D-Structure (Drug)^b^	(73, 74, 75, 76)
Glycosidase inhibition	α-Glucosidase I-II	Celgosivir	https://pubchem.ncbi.nlm.nih.gov/compound/60734section=2D-Structure (Drug)^b^	(77, 78)
		Castanospermine	https://pubchem.ncbi.nlm.nih.gov/compound/54445section=2D-Structure (Drug)^b^	(77, 78)
Chitosan-binding protein inhibition	Chitosan-binding domain of S glycoproteins	Chitosan polysaccharide	https://pubchem.ncbi.nlm.nih.gov/compound/71853section=2D-Structure (Drug)^b^	(79, 80)
		N-(-2-hydroxypropyl)-3-trimethylammonium chitosan	https://pubchem.ncbi.nlm.nih.gov/compound/102340638section=2D-Structure (Drug)^b^	(81)
Heparan sulfate-binding protein inhibition	Heparan sulfate-binding domain of S glycoprotein RBD	Heparan sulfate	https://pubchem.ncbi.nlm.nih.gov/compound/53477715section=2D-Structure (Drug)^b^	(82, 83)
		Unfractionated heparin,^b^ 16,000 Da (54 saccharide units)	https://pubchem.ncbi.nlm.nih.gov/substance/53790558section=2D-Structure (Drug)^b^	(82, 83)
		Tinzaparin,^b^ low-molecular-weight heparin (LMWH), 6500 Da (22 saccharide units)	https://pubchem.ncbi.nlm.nih.gov/compound/772section=2D-Structure (Drug)^b^	(82, 83)
		Dalteparin,^b^ LMWH, 6000 Da (20 saccharide units)	https://pubchem.ncbi.nlm.nih.gov/substance/53787074section=2D-Structure (Drug)^b^	(82, 83)
		Enoxaparin,^b^ LMWH, 4500 Da (15 saccharide units)	https://pubchem.ncbi.nlm.nih.gov/compound/772section=2D-Structure (Drug)^b^	(82, 83)
Inhibition of the interaction between S1-RBD and the ACE2 receptor	S Glycoprotein RBD	Kanamycin,^b^ aminoglycoside antibiotic	https://pubchem.ncbi.nlm.nih.gov/compound/6032section=2D-Structure (Drug)^b^	(84)
		Amikacin,^b^ aminoglycoside antibiotic	https://pubchem.ncbi.nlm.nih.gov/compound/37768section=2D-Structure (Drug)^b^	(84)
		Acarbose,^b^ polysaccharide	https://pubchem.ncbi.nlm.nih.gov/compound/41774section=2D-Structure (Drug)^b^	(84)
Inhibition of SARS-CoV-2 protease and RBD S glycoprotein	SARS-CoV-2 main protease and S glycoprotein RBD	Gentamicin,^b^ aminoglycoside antibiotic	https://pubchem.ncbi.nlm.nih.gov/compound/3467section=2D-Structure (Drug)^b^	(85)
Inhibition of S glycoprotein - ACE2 interaction	ACE2	Lividomycin, aminoglycoside antibiotic	https://pubchem.ncbi.nlm.nih.gov/compound/72394section=2D-Structure (Drug)^b^	(86)
Inhibition of host importin nuclear transport proteins	Host importin alpha/beta-1 nuclear transport proteins	Ivermectin,^b^ antiparasitic macrolide glycoside	https://pubchem.ncbi.nlm.nih.gov/compound/6321424section=2D-Structure (Drug)^b^	(87, 88)
Inhibition of RBD S glycoprotein - ACE2 interaction	S glycoprotein RBD	Digoxin,^b^ cardiac glycoside	https://pubchem.ncbi.nlm.nih.gov/compound/2724385section=2D-Structure (Drug)^b^	(89, 90)
		Ouabain,^b^ cardiac glycoside	https://pubchem.ncbi.nlm.nih.gov/compound/439501section=2D-Structure (Drug)^b^	(90)
Inhibition of SARS-CoV-2 protease and RBD S glycoprotein	SARS-CoV-2 main protease, RNA-dependent RNA polymerase and S glycoprotein RBD	Hesperidin,^b^ flavonoid glycoside	https://pubchem.ncbi.nlm.nih.gov/compound/10621section=2D-Structure (Drug)^b^	(91, 92, 93, 94)
		Rutin,^b^ flavonoid glycoside	https://pubchem.ncbi.nlm.nih.gov/compound/5280805section=2D-Structure (Drug)^b^	(85, 91, 92, 93, 94)
		Quercitrin,^b^ flavonoid glycoside	https://pubchem.ncbi.nlm.nih.gov/compound/5280459section=2D-Structure (Drug)^b^	(85, 91, 92, 93, 94)

a All suggested ways to COVID-19 therapy must be validated on clinical trials before their pharmacotherapy application.

b FDA-approved drug (https://www.fda.gov/drugs/drug-approvals-and-databases/drugsfda-data-files).

c Currently in clinical trials (ClinicalTrials.gov Identifier: NCT02257177).

Also focused on viral glycans of SARS-CoV-2 spike glycoprotein, it would be important to enhance the innate immunity by using recombinant human lectins. Exogenous administration of MBL in elderly humans can contribute to improve the immune response of patients. MBL gene polymorphisms were associated with susceptibility to severe acute respiratory syndrome infection, thus showing the importance of MBL in these viral infections (71, 72). Homozygous and heterozygous subjects for the variant A230 nucleotide (codon 54, exon 1) had increased susceptibility to SARS-CoV infection, compared with the homozygous G230 wild-type allele (71). This might be explained by reduced levels of functional MBL secondary to the A230 variant, thus losing immunological efficacy. Replacement protein strategy using human wildtype cDNA G230 MBL in patients during the early days of SARS-CoV-2 acute phase (pneumonia) can contribute to a milder evolution of disease (Table 1).

**Figure 5:**
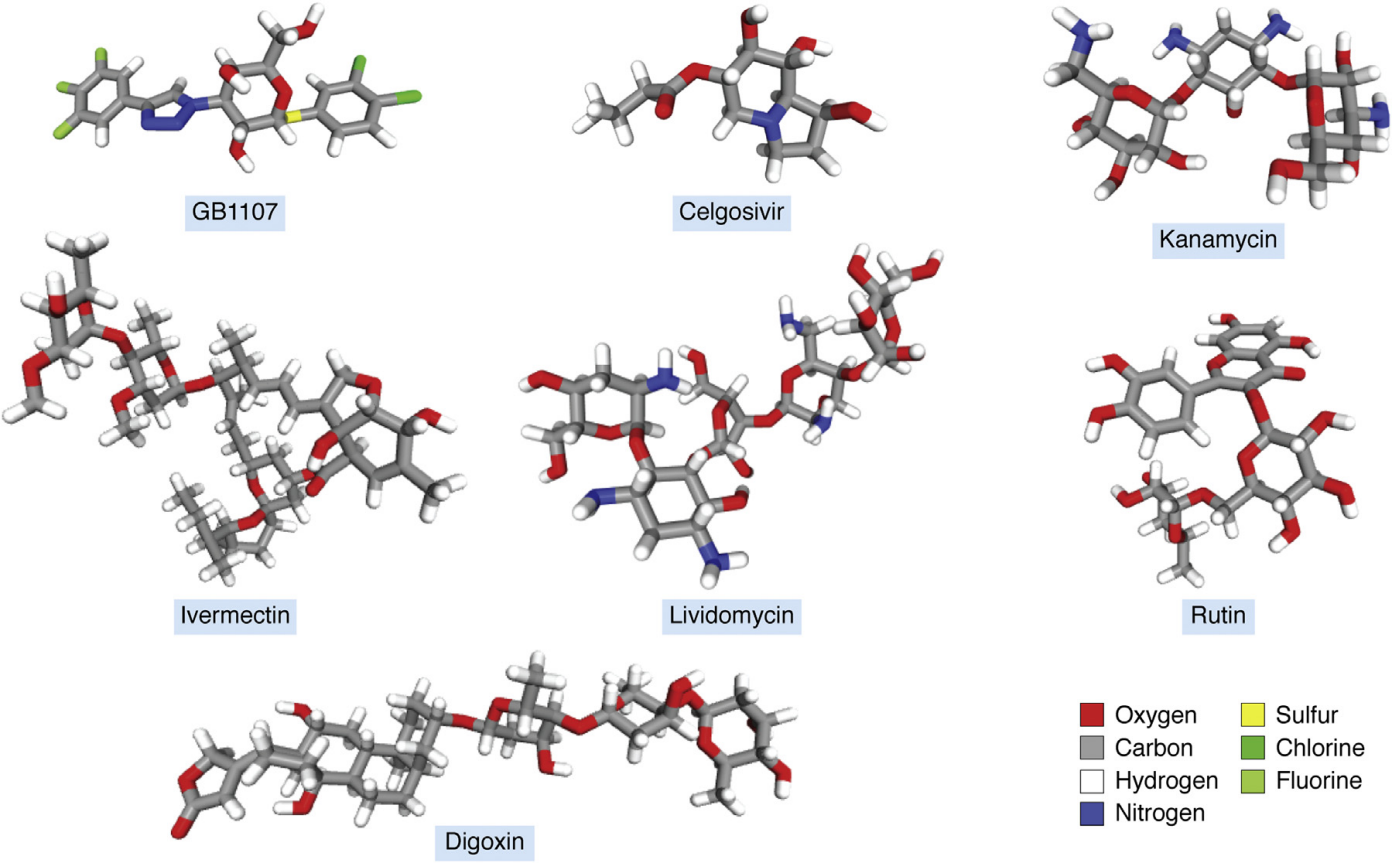
**Chemical structural conformation of potential therapeutic drugs**. Structural conformers presented as sticks were retrieved from PubChem (https://pubchem.ncbi.nlm.nih.gov).

Galectin 3 (Gal-3) is a member of the carbohydrate-binding protein family of human galectins. Gal-3 is a secreted lectin with potent proinflammatory effects that increase the production of interleukin 6 and tumor necrosis factor α, two cytokines with a critical role in cytokine storm–induced pneumonia causing fatal outcome in patients with COVID-19. Treatment with Gal-3 inhibitors looks promising in reducing the cytokine storm in SARS-CoV-2–infected patients. Some of the Gal-3 inhibitors such as GB1107, TD139, and belapectin are currently in clinical trials (73, 74, 75, 76) (Table 1 and Fig. 5).

Another interesting therapeutic tool as a mean to affect viral glycosylation are glycosidase inhibitors. Iminosugars are known inhibitors of the α-glucosidase I and II enzymes (two critical enzymes in the N-glycan biosynthesis) that effectively inhibited SARS-CoV-2 viral replication (77): Celgosivir, castanospermine, and UV-4 were reported to have this biological activity (Table 1 and Fig. 5). The focus here would be to reduce the host cell N-glycosylation pathway to control virus replication (78).

Several viruses (influenza virus, herpes simplex virus, human immunodeficiency virus, SARS-CoV-1, and MERS) use glycans to facilitate the initial interaction with host cells (95, 96), and SARS-CoV-2 also uses this approach. Focusing again into preventing SARS-CoV-2 interaction with host cell (and further entry for replication), several glycans and glycosides should be considered for specific therapies. Chitosan has shown a very important capacity in reducing SARS-CoV-2 infection through the N-(-2-hydroxypropyl)-3-trimethylammonium chitosan chloride compound (81). Since the fusion of viral membrane with host cell membrane is mediated by the S2 subunit of S glycoprotein, it is likely that the interaction of chitosan and derived chitosan molecules with the S2 subunit interferes with the fusion events, thus reducing incorporation of viral RNA into host cells (Table 1). Chitosan and related chitosan molecules are widely used as drugs for different therapeutic applications such as particulate systems for drug and vaccine delivery (79). These molecules are also used in tissue engineering as extracellular matrix because they are innocuous, nonimmunogenic, and nontoxic (80).

Another potential therapeutic molecule is HS, which works as a coreceptor (along with ACE2) for RBD binding. Indeed, cellular HS is required for efficient SARS-CoV-2 virus infection (39). Heparin and HS showed inhibitory capacity toward the S glycoprotein RBD ability to interact with different cell types. Heparan 3-*O*-sulfation (decisive for the anticoagulant activity of heparin) had slight effect on its inhibition of S glycoprotein binding, suggesting that inhibition by related heparan molecules is most likely charge dependent rather than anticoagulant activity dependent (39). Heparin and HS used in medicine are important drugs to reduce the SARS-CoV-2 infection (82, 83) (Table 1).

*In silico* studies for docking and molecular dynamics simulation processes have also predicted the interaction ability of S glycoprotein RBD with various molecules related to the field of glycobiology corresponding to repurposed Food and Drug Administration (FDA)-approved drugs (Table 1 and Fig. 5). Several aminoglycoside antibiotics such as kanamycin and amikacin and polysaccharides such as acarbose molecules showed important interaction with S glycoprotein RBD (84). RBD amino acids described to be involved in the interaction with amikacin (R403, E406, K417, Y453, and Y495) are interspersed with those interacting with HS (R346, F347, S349, N354, R355, K444, G447, Y449, Y451, and R466), thus suggesting to be all in the same glycan-binding pocket. A new advanced *in silico* drug discovery method for novel coronavirus (SARS-CoV-2) with tensor decomposition-based unsupervised feature extraction described gentamicin aminoglycoside antibiotic as candidate drug (85). Insights from a molecular mechanics–assisted structure-based virtual screening experiment showed lividomycin aminoglycoside antibiotic as a potential ACE2 inhibitor in the COVID-19 pandemic (86) (Table 1 and Fig. 5). Besides their predicted binding to important proteins for SARS-CoV-2 infection, the aminoglycoside antibiotics are also translational inhibitors and defensin releasers that mediate antiviral activity as immunity enhancers (97), supporting their relevance for COVID-19 therapy. Aminoglycosides were also proved to produce functional peptides from theta defensins (known as retrocyclins) that are active against HIV (98). Since antibiotics are used for treating the COVID-19 pneumonia, the aminoglycoside antibiotics could represent a therapeutic contribution against SARS-CoV-2 virus. These considerations should be evaluated in clinical trials.

Macrolides are molecules with a large macrocyclic lactone ring to which one or two deoxy sugars may be attached. Macrolide antibiotics did not show clinical evidence for antiviral properties on SARS-CoV-2 infection (99), and azithromycin did not improve clinical outcome when used together with hydroxychloroquine (100). Ivermectin (an antiparasitic macrolide) revealed broad-spectrum antiviral activity (101), also evidenced against SARS-CoV-2 in *in vitro* assays (87) (Table 1 and Fig. 5). Ivermectin inhibits the formation of the importin-α (IMPα) and IMPβ1 subunits as well as dissociates the IMPα/β1 heterodimer affecting the molecular transport to host nucleus and the viral replication (102). Multiple clinical trials using ivermectin are currently under development (88).

Repurposing of FDA-approved small molecule drug inhibitors of SARS-CoV-2 S protein and human ACE2 interaction through virtual screening approaches showed digitoxin cardiac glycoside to be a good therapeutic candidate drug (89, 103). Antiviral activity of digoxin and ouabain cardiac aminoglycosides against SARS-CoV-2 infection was recently demonstrated (90), thus suggesting that these cardiac aminoglycosides may be alternative treatments for COVID-19 with additional benefits for patients with cardiovascular disease (Table 1 and Fig. 5). Digoxin showed to be of most use in heart failure with reduced ejection fraction and in atrial fibrillation with rapid ventricular response for rate control, especially when associated with hypotension (104).

The identification of potential inhibitors of SARS-CoV-2 main protease using molecular docking studies revealed flavonoid glycosides as candidate molecules (105). Flavonoid glycosides are polyphenolic structures with covalent linkage of sugars, and hesperidin, rutin, and quercitrin were identified as ligands of SARS-CoV-2 main protease, RNA-dependent RNA polymerase, and S glycoprotein RBD (85, 91, 92, 93, 94) (Table 1 and Fig. 5). These repurposed FDA-approved drugs could protect against SARS-CoV-2 infection, where an important endothelial dysfunction probably associated to systemic complications is produced (106). Hesperidin stimulates production of nitric oxide in endothelial cells while improving endothelial function, thus reducing inflammatory markers of patients (107). Rutin and related flavonoid glycosides are vessel protectors that could help to control the endothelial dysfunction with potential antiviral activity. All together this information suggests that FDA-approved flavonoid glycosides could be useful drugs in the urgent therapeutic situation of COVID-19 patients with multisystemic complications.

## Outlook

Several viruses utilize the glycosylation machinery to enhance their infectivity, thus advising to consider therapeutic ways within the field of glycobiology. In this urgent pandemic situation, drug repurposing strategies such as application of widely existing approved drugs could be an ideal and rational approach to bring therapeutic products into market in quick times: we have here suggested repurposing of various FDA-approved drugs. Glycan antigens, anti-glycan antibodies, glycan-binding proteins, lectin inhibitors, polysaccharides, glycosidase inhibitors, and glycosides are all drugs to be considered regarding COVID-19 prevention and therapy. As mentioned above, all of these proposals would need appropriate medical review. Because the SARS-CoV-2 S glycoprotein RBD binding pocket for oligosaccharides is adjacent but separate from the ACE2-binding site, an original approach may be to find inhibitory molecules for both sites simultaneously, a condition where glycosides could do important contributions. In addition, the ability of glycosides to be ligands for more than one molecular target opens up the door to additive effects: simultaneous regulation of SARS-CoV-2 protease and RNA-dependent RNA polymerase activities, as well as the binding ability to S glycoprotein RBD, represents an attractive therapeutic contribution from innocuous flavonoid glycosides that demands further exploration for COVID-19 therapeutic purpose. For the next-generation drugs suggested here, biotechnological engineering making new probes to block the SARS-CoV-2 infection might be based in the essential glycobiological insight from glycosyltransferases, glycans, glycan-binding proteins, and glycosidases related to this pathology.

## Dedications

This review is dedicated to the health workers around the globe for their strong efforts fighting COVID-19 pandemic.

## Conflict of interest

The authors declare that they have no conflicts of interest with the contents of this article.
